# Bipolar Radiofrequency Ablation Does Not Result in Full-Thickness Articular Cartilage Penetration: An Ex Vivo Bovine Investigation

**DOI:** 10.1016/j.asmr.2022.03.002

**Published:** 2022-04-11

**Authors:** Anthony N. Khoury, Maxwell J. Krupp, Andrea M. Matuska, Darren J. Friedman

**Affiliations:** a From Arthrex, Naples, Florida; b New York Presbyterian Lower Manhattan Hospital, New York, New York, U.S.A.

## Abstract

**Purpose:**

To evaluate the depth of penetration of manufacturer-recommended bipolar radiofrequency (BRF) output in healthy hyaline cartilage.

**Methods:**

Two matched knees from a bovine specimen were harvested for immediate testing. BRF probes were used to treat the articular cartilage in a hydrated noncontact technique employing a 1-mm spacer on patellar, condylar, and trochlear surfaces. Two manufacturer-recommended ablate power settings were evaluated to analyze the effect of varying power outputs on the depth of penetration. Surfaces were randomized and treated with BRF ablate setting 3 (AB-3), 4 (AB-4), or left untreated as a control (12 grids each). Slices were extracted from treatment zones and subjected to fluorescein diacetate and propidium iodide viability stains and analyzed with confocal light microscopy. A general linear model was used to determine whether variables such as ablation setting, cartilage location, and side significantly influenced depth of penetration (DoP) and cartilage thickness (Minitab 19, Chicago, IL). When significance was noted (*P* < .05), a post hoc-Tukey test was used to investigate specific differences.

**Results:**

AB-3 had a 50.9% lower mean DoP than AB-4 (*P* = .006). The mean DoP was 237.9 ± 140.6 μm for AB-3 and 484.1 ± 267.0 μm for AB-4. Median DoP values were 243.2 ± 149.5 μm for AB-3, 51.2% lower than the 498.4 ± 286.0 μm for AB-4. The mean maximum DoP for AB-3 was 302.4 ± 167.8 μm, 50.6% lower than AB-4 value of 611.6 ± 299.1 μm. Analysis of the cartilage thickness confirmed there was no difference in overall cartilage thickness used for AB-3 versus AB-4 testing (*P* = .953).

**Conclusions:**

The RF probe ablate power setting AB-3 demonstrated significantly less articular cartilage depth of penetration than the AB-4 setting in a healthy bovine model.

**Clinical Relevance:**

Debridement of chondral lesions with plasma BRF is of clinical interest. The presented study adds basic science information for those considering performing this technique.

Surgical bipolar radiofrequency (BRF) devices use pulsed-energy modulations in conductive fluid environments to create a stable gas plasma field. The highly energized plasma contains significant energy to dissociate organic molecular bonds, such as those in human tissue.^1^ Volumetric resection of damaged or fibrillated cartilage performed using BRF occurs through ionization of molecules in the environment, as opposed to application of high-voltage thermal heat or mechanical shaver. Cartilage lesion borders are debrided and stabilized to prevent propagation at a lower temperature compared with conventional electrocautery devices.^2^ Several studies have illustrated that plasma ablation is an option for cartilage debridement when used during arthroscopic procedures.^3^, ^4^, ^5^, ^6^

Although BRF has been proven effective in arthroscopic procedures, some remain concerned about chondrocyte viability following exposure. Chondrocyte necrosis can occur without visual indication and is a critical component for evaluating technologies. BRF power and contact time typically used in arthroscopic environments have been simulated in vitro to evaluate depth of penetration (DoP) cell necrosis in healthy hyaline cartilage. Cell viability is quantified by confocal light microscopy and fluorescent dye staining (live/dead staining).^3^^,^^4^^,^^6^, ^7^, ^8^, ^9^ Significant variability in DoP resulting from BRF exposure exists in the literature due to inconsistent testing conditions, BRF devices used, ablation settings, application techniques, fluid-flow management, and specimen selection. In addition, the established work uses early first- and second-generation BRF devices.^3^^,^^4^^,^^6^^,^^7^^,^^9^^,^^10^ To date, there is limited available literature evaluating DoP resulting from exposure to modern plasma BRF devices.

The purpose of this study was to evaluate the DoP of manufacturer-recommended bipolar radiofrequency output in healthy hyaline cartilage. The authors hypothesized that lower ablation output would result in 200 to 400 μm of maximal DoP and demonstrate less DoP compared with a greater ablation power output.

## Methods

**Fig 1:**
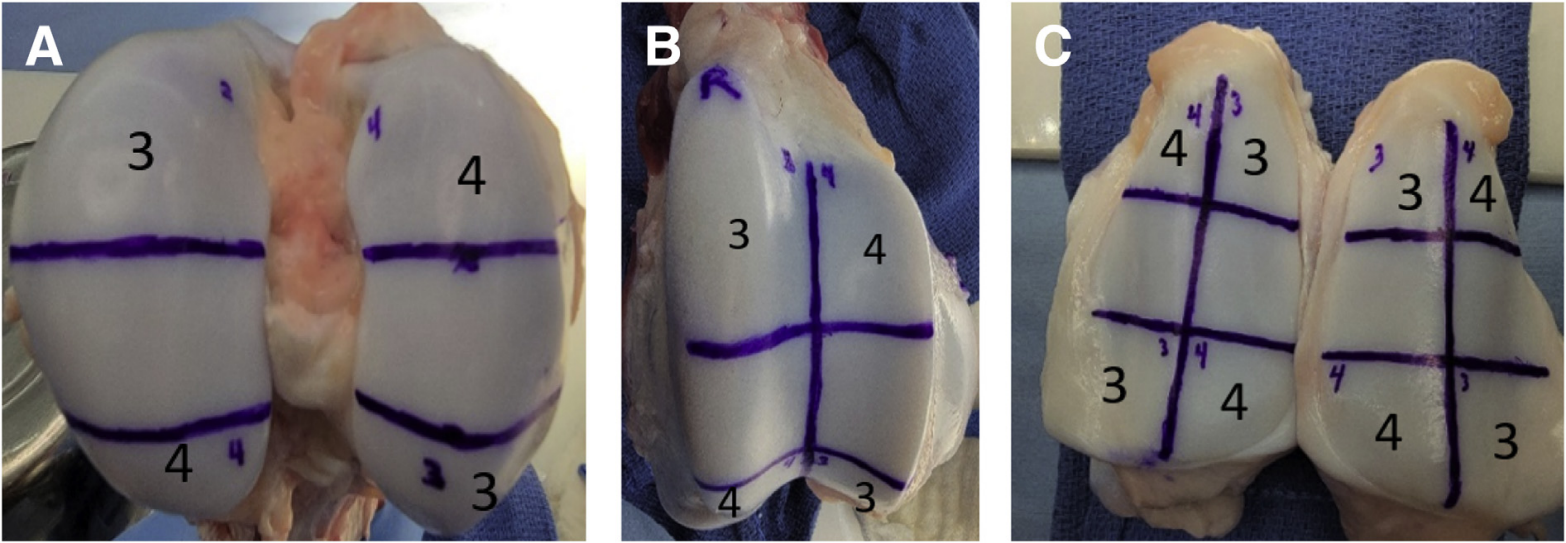
Example of treatment grids for (A) femoral condyle, (B) trochlea, and (C), and patella. Locations not indicated with an ablation setting (AB-3 or AB-4) were used for control analysis.

Institutional Animal Care and Use Committee approval was obtained before experimentation (#DB-667). DoP of BRF ablation was evaluated on the femoral condyle, trochlea, and patella of healthy bovine knee hyaline cartilage. A matched pair of knee specimens were obtained from a 7-month-old, 220-kg specimen euthanized for immediate use. No anatomic defects were observed on the matched knee specimens. A grid was drawn with a surgical marker and ruler on the medial and lateral location of each of the 3 anatomic sites. (Table 1) The grid was a chiral image for the matched knee specimens. Control spaces were centered on the distal condyle, central trochlea, and central patella to ensure similar cartilage thickness when comparing ablation settings. Overall, 24 testing spaces and 12 control spaces were assigned for evaluating 2 BRF ablation power (Fig 1). Once each specimen was marked, it was submerged in a basin filled with room-temperature saline.

**Table 1:** Treatment Grid Map

Femoral Condyle Grids			Trochlea Grids			Patella Grids		
Right	Lateral	Medial	Right	Lateral	Medial	Right	Lateral	Medial
Anterior	Ablate 3	Ablate 4	Superior	Ablate 3	Ablate 4	Superior	Ablate 4	Ablate 3
Distal	Control	Control	Central	Control	Control	Central	Control	Control
Posterior	Ablate 4	Ablate 3	Inferior	Ablate 4	Ablate 3	Inferior	Ablate 3	Ablate 4

Left	Lateral	Medial	Left	Lateral	Medial	Left	Lateral	Medial
Anterior	Ablate 3	Ablate 4	Superior	Ablate 3	Ablate 4	Superior	Ablate 4	Ablate 3
Distal	Control	Control	Central	Control	Control	Central	Control	Control
Posterior	Ablate 4	Ablate 3	Inferior	Ablate 4	Ablate 3	Inferior	Ablate 3	Ablate 4

**Fig 2:**
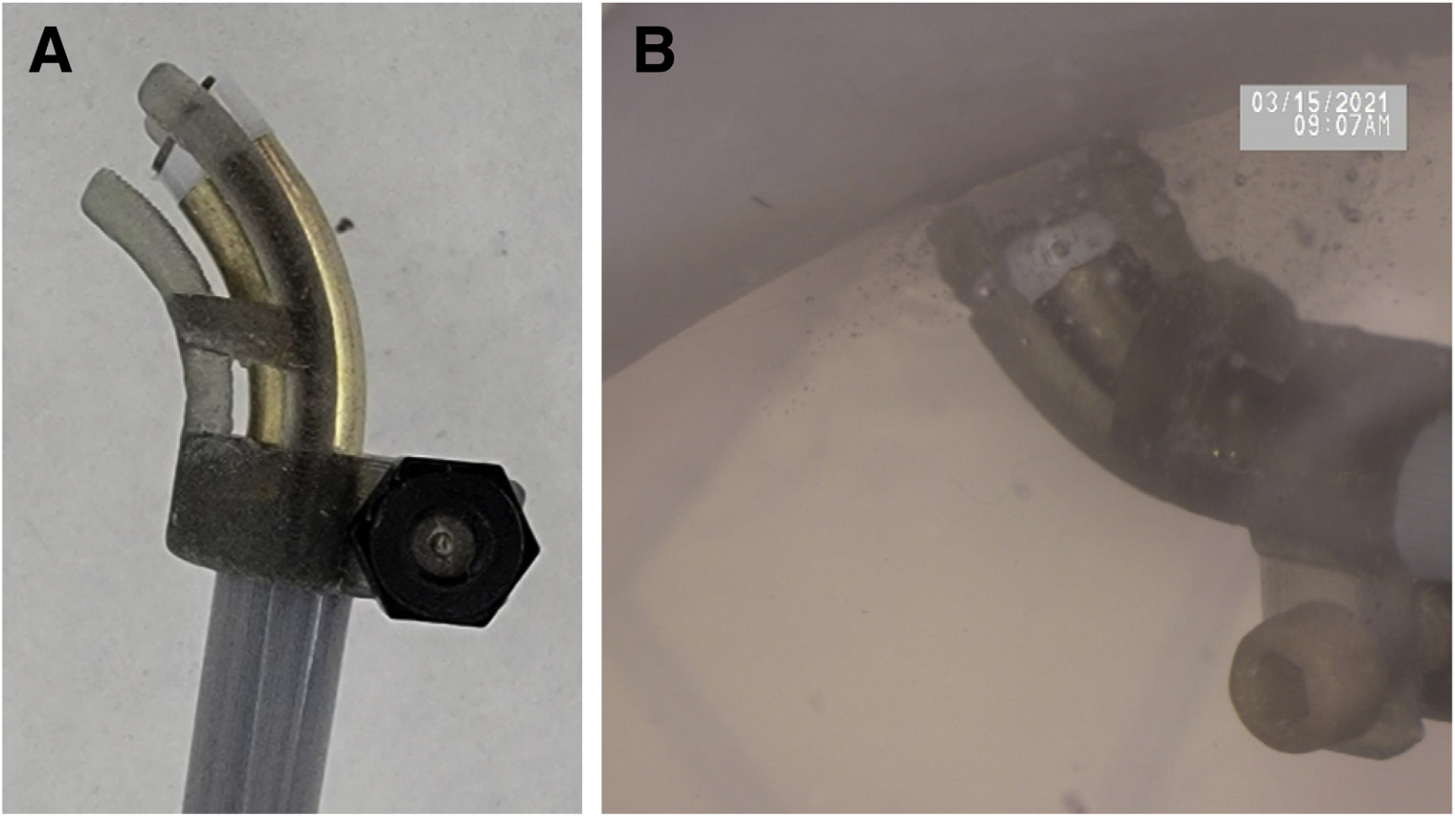
Customized spacer attached to the (A) bipolar radiofrequency probe and (B) in use.

Two ablation power settings were applied to evaluate DoP. Ablation setting 4 (AB-4) is the default setting of the BRF probe and ablation setting 3 (AB-3) is the manufacturer-recommended setting for ablation near sensitive tissue (Apollo MP50 and Synergy console, both Arthrex, Naples, FL). Probe aspiration was set to –350 mm Hg (within manufacturer-recommended range) using a Gast vacuum pump (Gast Manufacturing, Inc., Benton Harbor, MI). BRF ablation was applied to the specimens in linear strokes. A noncontact approach was standardized 1 mm from cartilage (±0.1 mm tolerance) using a customized 3-dimensional–printed spacer fixed to the BRF probe (Fig 2). The device was activated continuously and passed over the cartilage at a rate of 3 to 4 mm per second, allowing the plasma field to contact the target tissue. This technique was applied for both the AB-3 and AB-4 power settings. No deformation or spacing changes were noted throughout the evaluation.

Fresh saline bath was used for each specimen. Following treatment, specimens were placed in bench-stable MEM GlutaMAX (Gibco, ThermoFisher, Waltham, MA) and labeled accordingly. A suture was placed in right sided specimens for additional identification. Specimens were packaged on ice and shipped overnight to an external laboratory for analysis. (StageBio, Inc., Thurmont, MD).

Upon receipt, the samples were examined to confirm good condition and refrigerated at 4°C. Each specimen was trimmed to capture 1- to 2-mm thick sections, moving medial to lateral through the treatment grids. The sections were immersed in phosphate-buffered saline and subjected to fluorescein diacetate and propidium iodide (Sigma-Aldrich, St. Louis, MO) viability stains to identify living and dead chondrocytes. The sections were mounted to slides and imaged using a BZ-X810 All-in-One Fluorescence Microscope (Keyence, Itasca, IL).

**Fig 3:**
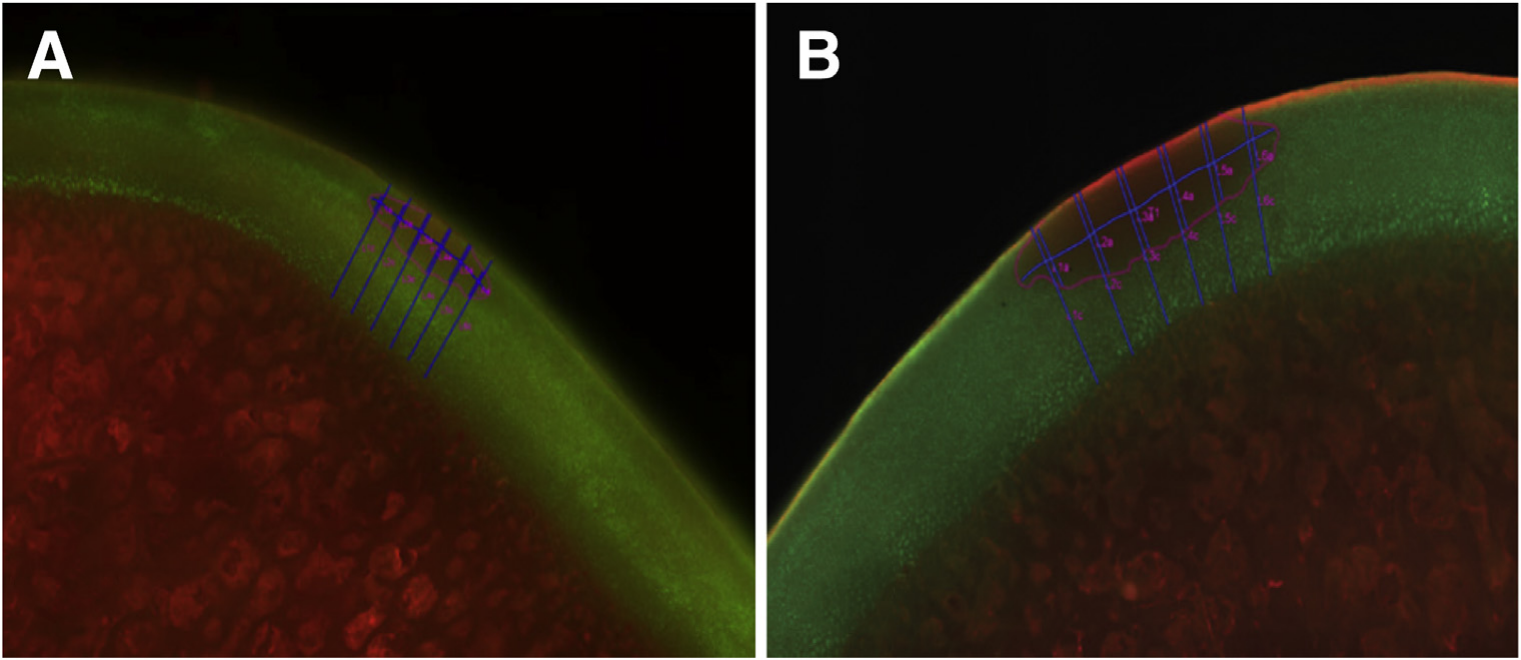
Depth of penetration measurement. (A) Cartilage thickness was measured at 6 locations in the BRF treatment zone perpendicular to the surface and base of the cartilage. (B) Ablation lesion depth was measured at 6 locations in the BRF treatment zone from the cartilage surface to the deepest point of cellular necrosis. (BRF, bipolar radiofrequency.)

A histomorphometry analysis was performed and images analyzed with Adobe (Adobe Systems, San Jose, CA). Lesion width was measured at the approximate location of maximum width and was traced along the coarse contour of the cartilage across the lesion. Cartilage thickness measurements (micrometers) were recorded at 6 locations throughout each treatment zone with approximate even spacing and perpendicular to the surface and base of the cartilage (Fig3A). At the same locations, 6 ablation lesion depth measurements (micrometers) were obtained, tracing from the surface of the cartilage to the deepest associated point of cellular necrosis (Fig 3B).

### Statistical Analysis

Mean and standard deviation along with 95% confidence intervals are reported. A general linear model was used to determine whether variables such as ablation setting, cartilage location, and side significantly influenced DoP and cartilage thickness measures (Minitab 19, Chicago, IL). When significance was noted (*P* < .05), a post hoc-Tukey test was used to investigate specific differences.

## Results

Twenty-four slices were harvested from the treatment sites (12 for AB-3 and 12 for AB-4). Treatment grids required approximately 3 to 4 linear passes, with each pass requiring approximately 3 seconds. Two treatment sites (one site for AB-3 and AB-4) were excluded from the analysis due to the inability to extract conclusively treated tissue. No macroscopic changes to the tissues were observed after treatment.

The customized spacer maintained consistent electrode-to-tissue spacing of 1 mm; however, the sloping geometry of the patella resulted in 2 instances of excessive DoP (966.5 μm and 1228 μm maximum depth points), which were both included in the analysis. In both cases, the electrode came in contact with the tissue, resulting in greater DoP. These outliers impacted the DoP results of AB-4 contributing to a greater overall mean DoP.

**Fig 4:**
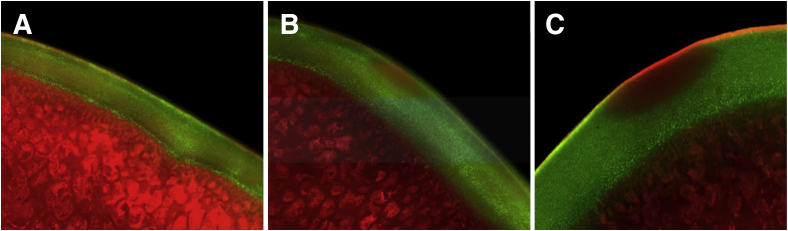
Representative images showing chondrocyte viability. (A) Control. (B) After exposure to power setting Ablate 3 (AB-3). (C) After exposure to power setting Ablate 4 (AB-4).

Microscopic images captured ablation treatment DoP. The control sites exhibited healthy cell structure with little to no cellular death (Fig 4A), whereas distinct oval thermal margins were observed following exposure to power setting AB-3 (Fig 4B) and AB-4 (Fig 4C). No cavitation was noted on the chondral surfaces of treated tissues. No difference in cartilage thickness used for AB-3 vs AB-4 testing was observed (*P* = .953). The condylar cartilage was significantly thinner (833 ± 208 μm) than both patellar (1366 ±118 μm) and trochlear cartilage (1352 ± 503 μm) (*P* = .021).

Detailed mean, standard deviation, and 95% confidence intervals for penetration depth resulting from AB-3 and AB-4 power settings is reported in Table 2. Mean DoP for AB-3 was 237.9 ± 140.6 μm, 50.9% lower than AB-4 which measured 484.1 ± 267.0 μm (*P* = .006). Median DoP for AB-3 was 243.2 ± 149.5 μm and 498.4 ± 286.0 μm for AB-4 (*P* = .007). Average maximum DoP for AB-3 was 302.4 ± 167.8 μm, 50.6% lower than 611.6 ± 299.1 μm for AB-4 (*P* = .002). Cartilage side or location did not significantly influence BRF penetration depth of AB-3 or AB-4 power settings for mean (*P* = .444, *P* = .071), median (*P* = .439, *P* = .079) or maximum (*P* = .242, *P* = .057), respectively.

**Table 2:** Mean ± Standard Deviation and 95% Confidence Intervals for All Data

	Condyle	Trochlea	Patella	Overall
Mean				
Ablate 3	236.7 ± 119.3 (–59.6, 533.0)	191.4 ± 157.8 (–59.7, 442.4)	285.3 ± 160.1 (30.5, 540.1)	237.9 ± 140.6 (143.4, 332.3)
Ablate 4	405.8 ± 247.0 (12.7, 798.8)	346.5 ± 154.2 (101.1, 591.9)	771.9 ± 234.2 (190.2, 1353.6)	484.1 ± 267.0 (304.7, 663.4)
Median				
Ablate 3	244.3 ± 125.3 (–66.8, 555.5)	192.8 ± 170.7 (–78.9, 464.4)	292.8 ± 168.1 (25.3, 560.4)	243.2 ± 149.5 (142.8, 343.7)
Ablate 4	408.0 ± 273.6 (–27.3, 843.3)	360.7 ± 156.8 (111.2, 610.1)	802.0 ± 259.0 (160.2, 1444.7)	498.4 ± 286.0 (306.2, 690.5)
Max				
Ablate 3	323.6 ± 176.9 (–115.9, 763.2)	241.4 ± 163.3 (–18.5, 501.3)	347.5 ± 195.3 (36.7, 658.2)	302.4 ± 167.8 (189.7, 415.1)
Ablate 4	542.0 ± 205.4 (215.2, 869.8)	429.1 ± 190.2 (126.5, 731.7)	947.6 ± 290.2 (226.6, 1668.6)	611.6 ± 299.1 (410.6, 812.5)

## Discussion

This ex vivo investigation of BRF cartilage ablation revealed the lower power setting, AB-3, resulted in significantly less DoP (mean: 237.9μm) compared with AB-4 (mean: 484.1μm). In addition, the median and maximum DoP for AB-3 was significantly lower than the AB-4 power setting by approximately 50%. Location of BRF exposure (femoral condyle, trochlea, patella) did not significantly affect DoP for either ablation power setting. Findings confirm the proposed hypothesis that lower ablation power settings will have decreased articular cartilage DoP.

Iatrogenic articular cartilage damage, informally termed arthroscrapes, is an unintended consequence of arthroscopic procedures.^11^ Considering the high volume of chondral lesions encountered,^12^^,^^13^ the frequency and physiological effects of arthroscrapes should be quantified. A review of surgeon-published instructional arthroscopic procedure videos available online revealed a 70% incidence of iatrogenic damage, suggesting reporting may be underrepresented in the literature.^14^ Preservation of healthy cartilage and stabilization of cartilage lesion borders are both critical to patient outcomes and can potentially delay the need for additional surgery. There is a lack of recent in vitro investigations using modern plasma BRF systems and probes. Therefore, this investigation sought to quantify cell viability and penetration depth of a modern plasma BRF probe and manufacturer-recommended power settings.

Critical limitations of cartilage debridement with mechanical shaver include difficulty creating a smooth surface and stable transition zone resulting in possible over-resection of healthy cartilage.^2^^,^^15^ Whereas monopolar radiofrequency probes deliver energy through one electrode, giving off high heat during the process, BRF probes use a plasma field generated between the 2 electrodes to accomplish tissue resection. The plasma field is a result of ionization of the fluid medium between the electrodes, a process different from the original thermal energy designs.^2^^,^^15^ Therefore, the plasma field characteristic of modern BRF probes allows for precise debridement at lower surrounding temperatures. The device used in this investigation delivered a maximum power of 232 Watts with a rated load of 70 ohm for setting AB3 and 255 Watts with a rated load of 100 ohm for setting AB4.

Evaluation of chondrocyte response to BRF has been reported with mixed results. Retrospective clinical analyses describe low complications and successful treatment of chondral lesions.^5^^,^^15^ In addition, treatment with radiofrequency energy has been shown to be more efficacious compared with mechanical debridement.^16^ An early report from Edwards et al.^17^ caution the use of bipolar energy due to chondrocyte damage up to 2,810 μm. Caffey et al.^7^ compared 5 RF systems using contact or noncontact techniques and discovered DoP resulting in cellular necrosis ranging from 333 to 1242 μm. Wang et al.^6^ examined the effects of coagulation and ablation BRF modes at varied-power settings and concluded high ablation setting resulted in lower thermal radiation injury compared with both coagulation and low-power ablation settings (295.2 μm, 1147.3 μm, and 1217.2 μm means, respectively). The average maximum DoP of AB-3 in the presented study is comparable with previous investigations,^6^^,^^7^ whereas the higher power setting, AB-4, demonstrated increased maximum values. Average median DoP for both settings was presented to control for possible outliers and was determined to be less than previous studies.^7^^,^^9^^,^^10^

Amiel et al.^3^ found BRF probes were capable of well-controlled debridement with a defined margin of cellular death approximately 100 to 200 μm deep when the device was used in a light-contact approach. The measurement technique used in their investigation differs from other live/dead studies. The study measures maximal DoP from the bottom of the excavated tissue site to the bottom of the cellular death zone. True DoP may be underestimated because the additional tissue death occurring from debridement is ignored. In contrast, there was no evidence of tissue cavitation or loss found at either setting in our study. A review of other literature found that lower margins of BRF DoP tend to fall between 200 and 350 μm.^4^^,^^6^^,^^8^ In addition, DoP can vary due to differing surgical techniques, power outputs, and product designs. The results of the presented study add to the foundation of historical literature evaluating BRF ablation penetration depth and cell viability and provide a quantitative assessment of a modern device.

This study attempted to minimize variability through several means. A bovine specimen was selected to best mimic human cartilage, in regards to both thickness and biomechanical properties.^18^, ^19^, ^20^ Both of the BRF probe settings that were evaluated are recommended by the manufacturer for use in arthroscopic procedures. AB-3 is recommended for ablation near sensitive tissue due to its lower wattage while still maintaining a stable plasma field. Ablation was performed using recommended manufacturer aspiration settings to simulate a real clinical scenario. The customized spacer enabled the surgeon to maintain a noncontact spacing of approximately 1 mm. This noncontact approach attempted to mimic techniques that similar studies employed as well as replicate clinical usage.^6^^,^^7^ The acute harvest of specimen tissues, treatment, and lab analysis were all controlled to maintain cellular viability.

### Limitations

The presented study is not without limitations. This basic science investigation employed 2 bovine knees in an ex vivo setting. Further information regarding BRF ablation DoP can be attained with additional samples and cartilage mediums. BRF application was continuous over the testing grid to ensure sufficient exposure for cell viability analysis. This is not consistent with the surgical technique and it is unlikely a cartilage lesion would be exposed to a similar duration of exposure. In addition, this basic science investigation of DoP chondrocyte viability did not evaluate a clinical benefit of the technique.

Assessment of chondrocyte viability using live/dead staining is an inherent limitation. The tissues were treated and analyzed in an approximate 36-hour period. Kaplan et al.^21^ found that chondrocyte viability can change depending on time of evaluation and thermal exposure rates, illustrating that proteoglycan synthesis is possible 1 week after exposure. The live/dead staining methodology provides a snapshot of the cell viability postexposure and is not representative of regenerative potential.

This study assessed DoP of one BRF device manufacturer. Future studies evaluating DoP of recommended BRF ablation operating settings would provide useful information for surgeons using other BRF systems.

## Conclusions

The RF probe ablate power setting AB-3 demonstrated significantly less articular cartilage DoP than the AB-4 setting in a healthy bovine model.

## Supplementary Material

ICMJE author disclosure forms
